# Revisit to Endolymphatic Duct Identification Using Middle Cranial Fossa Dural Plate: A Novel Technique

**DOI:** 10.7759/cureus.73954

**Published:** 2024-11-18

**Authors:** Gautham S., Prasad K. C., Induvarsha G., Venkateshu K. V., Charuvi Guttal, Bosco L Suriya, Hithyshree N.

**Affiliations:** 1 Otolaryngology - Head and Neck Surgery, Sri Devaraj Urs Academy of Higher Education and Research, Kolar, IND; 2 Otorhinolaryngology - Head and Neck Surgery, Sri Devaraj Urs Academy of Higher Education and Research, Kolar, IND; 3 Anatomy, Sri Devaraj Urs Academy of Higher Education and Research, Kolar, IND

**Keywords:** cadaver dissection, endolymphatic duct, endolymphatic duct blockage surgery, endolymphatic sac decompression, meniere’s disease, middle cranial fossa dural plate

## Abstract

Objectives: Surgical treatments for Ménière's disease differ in efficacy. Endolymphatic duct blockage (EDB) is favored for its minimal risk and ability to preserve hearing. One of the main challenges in the technique is the difficulty in accurately identifying the endolymphatic duct (ED). The aim of the study is to see the feasibility of identifying the ED opening using a middle cranial fossa (MCF) dural plate as a reference point in a wet temporal bone and to measure the angle between the ED opening and the MCF dural plate which in the future can simplify the procedure of ED blockage.

Materials and methods: This prospective observational study, conducted from February to April 2024 at R L Jalappa Hospital, involved dissecting 20 wet temporal bones. A complete cortical mastoidectomy exposed the MCF dural plate, semicircular canals, sigmoid sinus, and sinodural angle. Using Donaldson line ES, through blind controlled drilling the operculum of the vestibular aqueduct was identified. The angle between the ED opening and the MCF dural plate was measured using GNU Image Manipulation Program (GIMP) software (Free Software Foundation Ltd., Boston, MA, USA). A tangential line from the MCF dural plate to the ED opening was drawn using the same software and the line was named the “GauthamaPrasads” (GP) line.

Results: The study documented angles between the ED opening and the MCF dural plate, which ranged from 36.00° to 45.99° (mean angles from 20 temporal bones provided).

Conclusion: The angle of 36.00° to 45.99° between the MCF dural plate and ED was consistently observed, thus proving that the MCF dural plate is a reliable anatomical landmark for surgeons for identification of ED. Accurate identification of the ED was feasible using this new anatomical landmark. These findings provide valuable anatomical insights that can aid in more efficient and accurate surgical interventions involving the ED.

## Introduction

The nonsensory elements of the endolymph-filled closed membrane labyrinth are the endolymphatic duct (ED) and the endolymphatic sac (ES). Through the vestibular aqueduct (VA), ED travels to the ES from the utricular and saccular ducts. From the external aperture, ES travels via the distal VA to terminate in the posterior cranial fossa epidural space [1]. Ménière's disease (MD) pathogenesis is due to ES dysfunction, which maintains the inner ear hydrostatic pressure and endolymph homeostasis [2].

MD is associated with structural abnormalities in the inner ear and fluid accumulation, known as endolymphatic hydrops. In endolymphatic hydrops, the perilymph, which envelops and fills the bony labyrinth decreases in volume while the endolymph which fills the membrane labyrinth increases in volume. Recurrent episodes of spontaneous usually rotational vertigo, sensorineural hearing loss, tinnitus, and a sensation of fullness or pressure in the affected ear are the hallmarks of MD. This condition often persists for several decades [3].

Management of the acute phase of MD primarily involves symptomatic treatment. Vestibular suppressants are widely recognized for their efficacy in mitigating acute episodes of vertigo, possessing diverse effects such as anticholinergic properties, anti-emetic activity, and vestibular sedation. In the long-term management of MD, strategies encompass adopting a low-sodium diet and administering diuretics during the post-acute phase [4].

Several surgical techniques have been devised to reduce the symptoms of MD, ranging in degree of invasiveness. Portman devised a technique for “decompressing” the ES by extracting the bone from the posterior cerebral fossa in order to relieve the symptoms of MD [5]. ESD includes a mastoidectomy and wide decompression of the ES [6]. The Donaldson line is used to estimate the location of the ES based on the prominence of the horizontal semicircular canal [7]. Surgeries on ES remain controversial as, many studies comparing ES surgery with sham procedures (either mastoidectomy or insertion of ventilation tubes), found no significant differences between the treatment and control groups. So, the efficacy of ESS remains questionable. Thus, a novel technique was proposed for alleviating symptoms associated with MD, by blocking the ED in order to reduce the endolymphatic volume within the inner ear. This procedure, known as ED blockage (EDB), involves placing a clip on the ED to isolate the ES from the remainder of the inner ear. The advantages of this technique include its permanent effect and its preservation of labyrinthine and inner ear functionality [8]. It needs identification of the ED opening without violation of the vital structures. Even though the Donaldson line is used to identify the ES, no specific landmark is used to identify the ESD opening. The main aim of the study is to see the feasibility of identifying the ED opening using the middle cranial fossa (MCF) dural plate as a reference point in a wet temporal bone by cadaveric microscopic dissection. This study also aims to measure the angle made while opening ED from the MCF dural plate. A line drawn by using that angle made from MCF towards the ED is named GP line, which is GauthamaPrasad’s line, which makes the identification of ED opening a non-tedious process.

## Materials and methods

This is a prospective observational study done on wet cadaveric temporal bones. The study was approved by the Central Ethics Committee with CEC number SDUAHER/KLR/R&D/CEC/S/PG.26/2024-25. The study period was taken from February 2024 to April 2024. We obtained 20 cadaveric temporal bones from the Department of Anatomy of Sri Devaraj Urs Medical College, Tamaka, Kolar. The study was done in the Temporal bone dissection lab of the Department of Otorhinolaryngology - Head and Neck Surgery, R L Jalappa Hospital, Tamaka, Kolar. Twenty wet cadaveric temporal bones were taken for dissection. They were mounted on temporal bone holders and fixed securely. Marathon M3 champion dental micromotor drill was used for dissection. After removing the soft tissues on the bone and exposing the temporal along with the landmarks, i.e., mastoid tip, and the spine of henlae, a complete cortical mastoidectomy was performed. MCF dural plate, three semi-circular canals, sigmoid sinus, and sinodural angle were delineated. Pre-sigmoidal dural plate was drilled for the complete exposure of the ES. Drilling was continued till the posterior semi-circular canal without violating its integrity. Posteriorly the bony exposure was done up to the level of the sigmoid sinus or even over the sigmoid sinus, however, a plate of bone (Bill’s Island) was preserved in order to compress the sigmoid sinus along the posterior fossa dura. At this point, we used Donaldson’s line to identify the ES, and with meticulous drilling of bone over the sac the operculum of the VA was identified. At this point, a photograph of the dissected specimen is taken keeping in mind the distance and angulation of all the specimens with the microscope is the same. The angle made by the ED opening with the MCF dural plate is documented using GNU Image Manipulation Program (GIMP) software (Free Software Foundation Ltd., Boston, MA, USA) and a tangential line is drawn from the MCF dural plate to the ED opening using the documented angle, this line is referred to as the GP line. This line can be used as a reference point for future surgeries so that aimless searching of the VA is avoided. The angle made by the ED opening with the MCF dural plate was tabulated and analyzed. We analyzed this data for a better understanding of the anatomy and a faster and more efficient method of locating the ED opening.

Statistical analysis

The data were presented as a number of specimens and percentages. The data were tabulated and interpreted.

## Results

**Figure 1:**
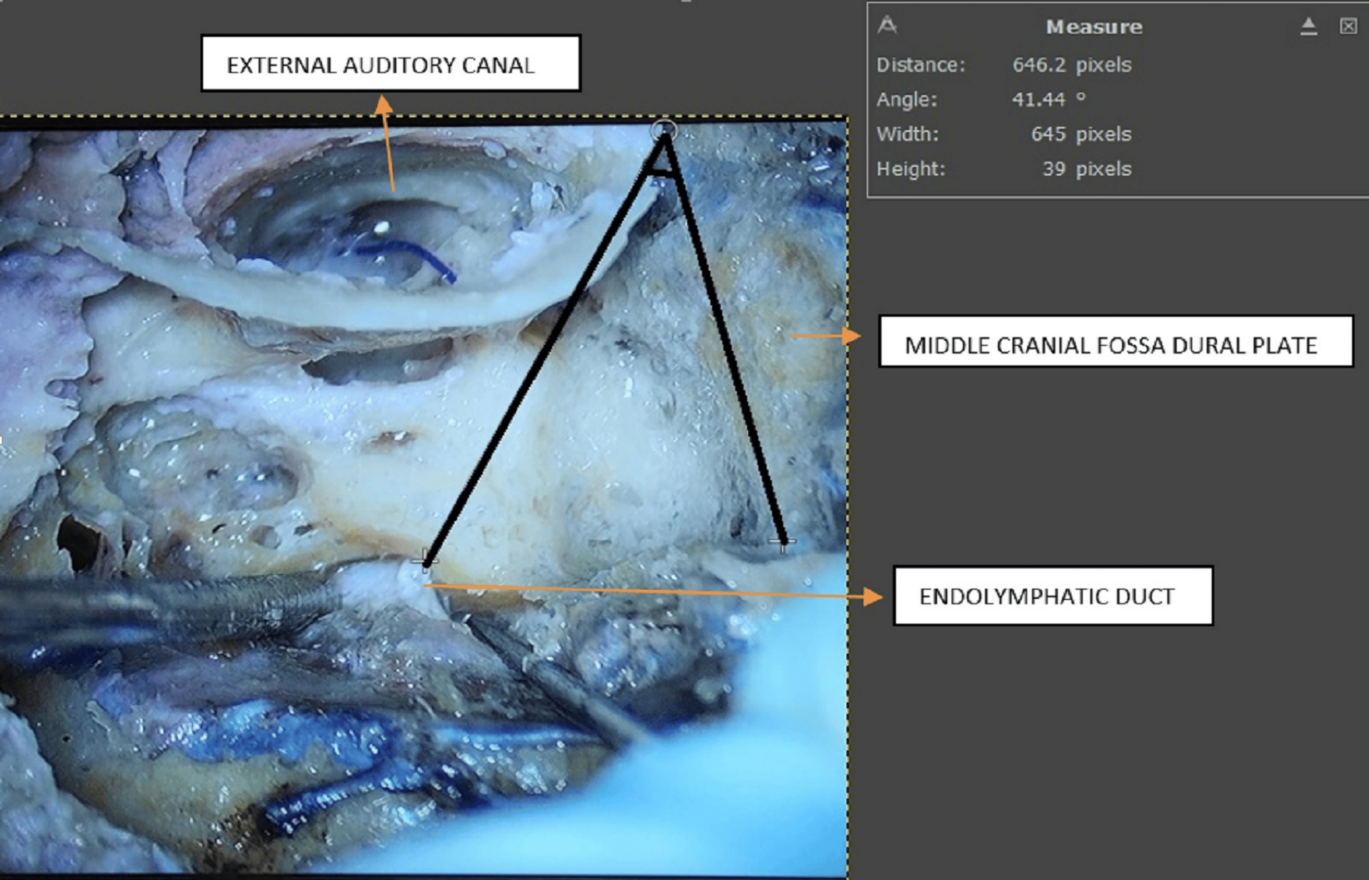
Line drawn from middle cranial dural plate to ED making an angle of 41.44° ED: endolymphatic duct

**Figure 2:**
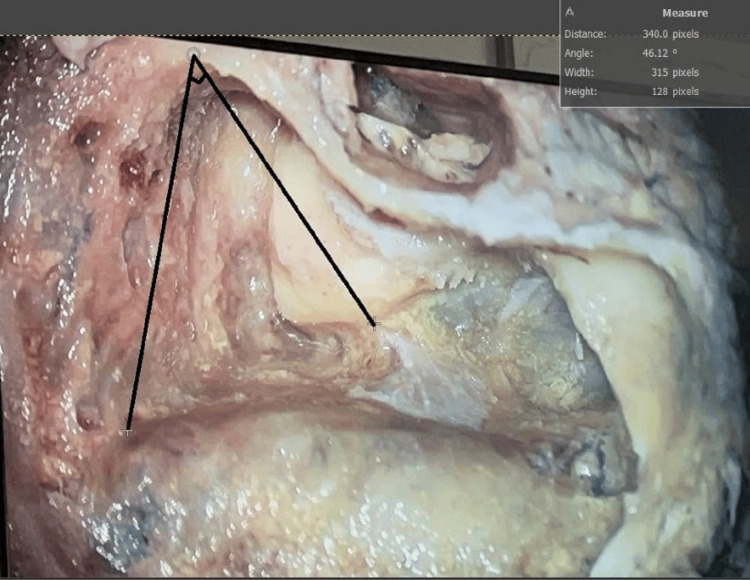
Line drawn from middle cranial dural plate to ED making an angle of 46.12° ED: endolymphatic duct

**Figure 3:**
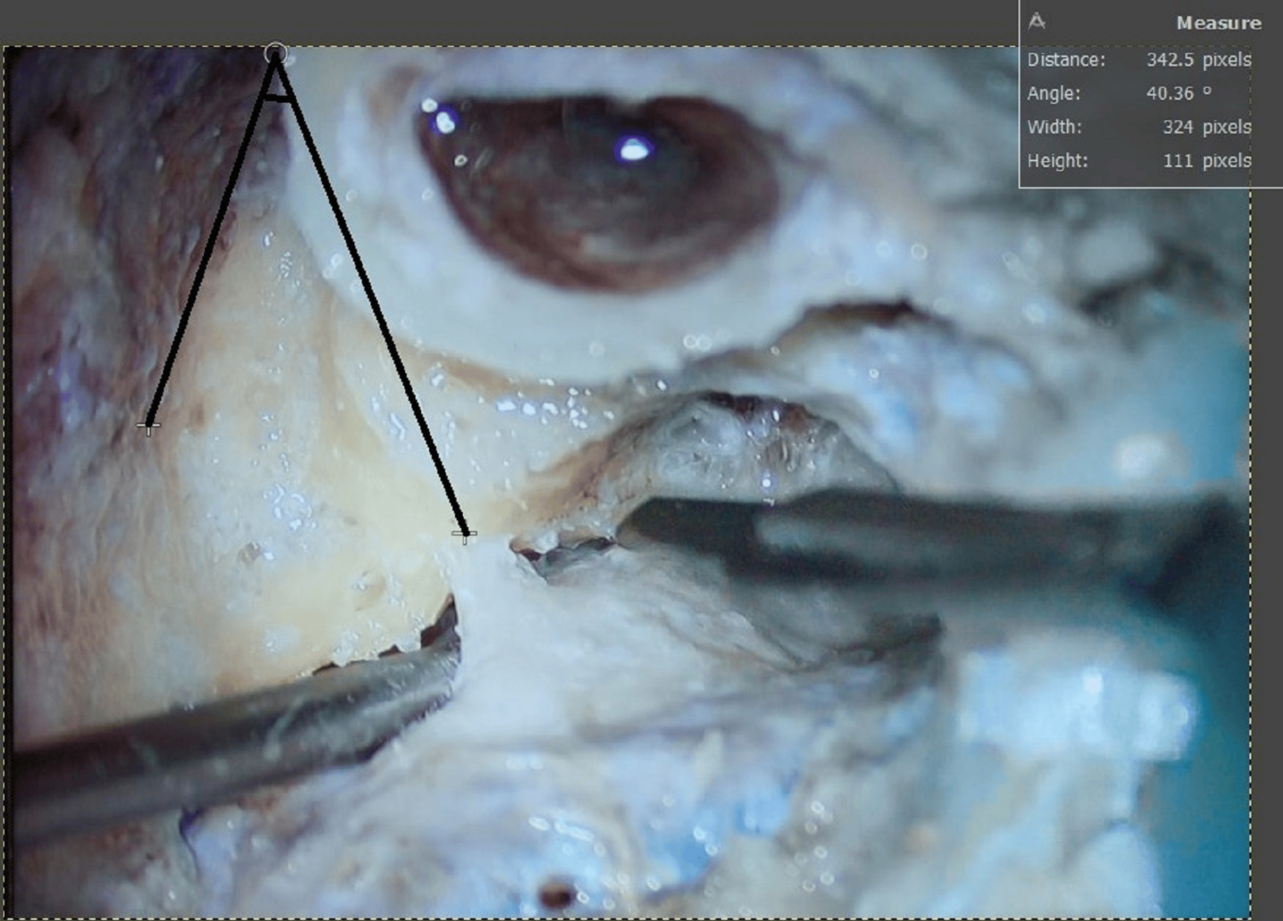
Line drawn from middle cranial dural plate to ED making an angle of 40.36° ED: endolymphatic duct

The study involved dissecting 20 wet temporal bones, out of which seven were right ear bones and 13 were left ear bones. In all 20 bones, a complete cortical mastoidectomy was done. All three semicircular canals were delineated. Drilling was continued till there was an eggshell bone over the sigmoid sinus, and the middle cranial dural plate was clearly defined. Donaldson line was used to identify the ES and with meticulous drilling, ESD opening was identified. A tangential line was drawn from the MCF dural plate at its highest point where it meets the posterior wall of EAC, to the ESD opening called as GP line (Figures 1-3), and the angle made by the ED opening from the MCF was documented. The results, summarized in Table 1, show the distribution of these angles across different ranges.

**Table 1:** Range of angles made by ED opening with MCF dural plate (degrees) ED: endolymphatic duct, MCF: middle cranial fossa

Angle range (°)	Number of temporal bones	Percentage (%)
35.00–35.99	2	10
36.00–36.99	4	20
37.00–37.99	1	5
38.00–38.99	2	10
39.00–39.99	2	10
40.00–40.99	3	15
41.00–41.99	3	15
42.00–42.99	4	20
43.00–43.99	1	5
44.00–44.99	0	0
45.00–45.99	2	10

This table categorizes the angles into ranges, providing a clearer overview of the distribution of angles observed in the study. The angles made by the ED opening ranged from 35.00° to 45.99°. The most frequent angle ranges observed were 36.00°-36.99° and 42.00°-42.99°, each accounting for 20% of the temporal bones dissected. Other significant angle ranges included 40.00°-40.99° and 41.00°-41.99°, each observed in 15% of the cases. The least frequent ranges were 37.00°-37.99°, 38.00°-38.99°, 39.00°-39.99°, and 43.00°-43.99°, each accounting for 5% to 10% of the cases. No angles were observed in the range of 44.00°-44.99°. The minimum angle formed by ED opening with MCF dural plate is 35.34°. The maximum angle formed by ED is 45.67°.

**Figure 4:**
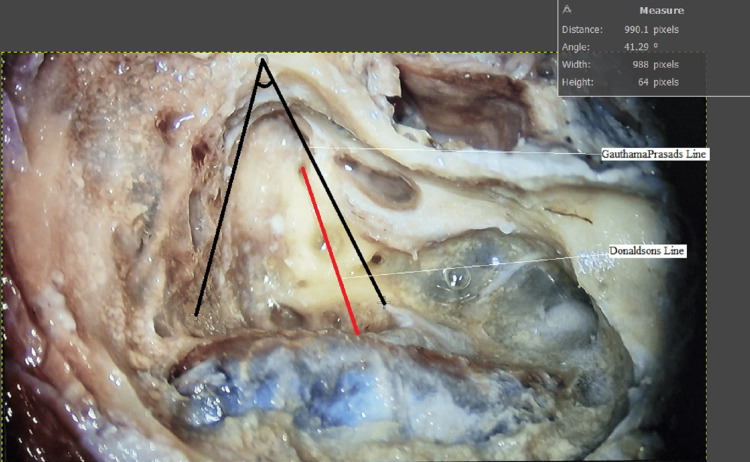
Relationship of Donaldson line and Gauthamaprasads line

The majority of the angles clustered around the 36.00° to 42.99° range, indicating a central tendency in the observed data. This clustering suggests a common anatomical alignment of the ED opening relative to the MCF dural plate in the studied temporal bones. The study successfully identified the angle ranges made by the ED opening relative to the MCF dural plate, with the most common angles falling between 36.00° and 42.99°. These findings provide valuable anatomical insights that can aid in more efficient and accurate surgical interventions involving the ED. GP line is more inferiomedially located compared to that of the Donaldson line (Figure 4), which further states that the endolymphatic opening is more inferiomedially located compared to that of the Donaldson line.

## Discussion

The ES was first described by Antonio Cotugno in his well-known dissertation “De Aquaeductibus auris humanae internae (On the Aqueducts of Human Internal Ear),” which was published in the year 1760 [9]. The ES is placed partially in the dura layers of the posterior cerebral fossa and partially within the petrous bone, which is covered by a thin piece of bone known as the operculum. The sac is located in a depression known as the foveate impression on the posterior aspect of the petrous bone. Within the dura mater, the extraosseous part of the ES projects laterally toward the sigmoid sinus and medially toward the jugular bulb. ES is located behind the posterior semicircular canal. The utricular duct joins the ED emerging from the saccule. The ED dilates proximally to the endolymphatic sinus. The vestibule's posterior wall has a depression where the sinus is situated. The sinus narrows and transforms into the ED as it enters the VA of the petrous bone. Within 1 mm of the vestibular opening, the duct narrows to form the ED's isthmus, which is the narrowest section with a diameter of 0.3 mm. Within the bony VA, the ED bends to take a lateral and inferior path after continuing in a posterior direction [5]. VA with concomitant dilatation of the endolymphatic sinus was described by the Italian Carlo Mondini in 1791 during an isolated temporal bone dissection of a deaf eight-year-old boy [9].

The ES retains the inner ear hydrostatic pressure and endolymph homeostasis, its dysfunction leads to the pathogenesis of MD [2]. MD is linked to anatomical abnormalities of the inner ear, that is endolymphatic hydrops. In endolymphatic hydrops, the perilymph, which envelops and fills the bony labyrinth decreases in volume while the endolymph which fills the membrane labyrinth increases in volume [3].

The three main symptoms of Ménière's illness are vertigo, deafness, and tinnitus or aural fullness. These symptoms are all intermittent and vary in severity. Even after 150 years since Ménière first described the disease, the true etiology and pathophysiology are not entirely understood even today which makes the management of the disease a tedious process. Thus, most of the initial management of the disease seemed to be empirical and no medical treatments appear to preserve long-term hearing. The acute medical management of MD includes vestibular suppressants and antiemetic medication. The chronic management of MD includes lifestyle adjustments like avoidance of triggers, salt restriction, usage of diuretics, aminoglycoside ablation, steroids, vasodilators, and complementary and alternative medicines. The medical modalities of treatment can control the disease in about 80% of the patients and not cure it completely [10].

If medical management fails, surgical management is preferred. To alleviate the symptoms of MD, a number of surgical techniques with differing degrees of invasiveness have been devised; however, their efficacy is questionable [11]. ESD has remained the surgery most commonly performed because of its conservative effect on hearing and low morbidity. However, its results are widely variable with a complete vertigo control rate. According to the meta-analysis by Sood et al., when mastoid shunts (with and without Silastic) were used, complete or substantial vertigo control was achieved in 75.7% (95% CI, 69.8%-81.2%) of patients, and postoperative hearing was stable or improved in 71.4% of them [12]. With ES decompression alone, postoperative substantial vertigo control was achieved in 79.3% of patients, and postoperative hearing was stable or improved in 72.8% of patients.

ES surgery was compared to a sham operation (mastoidectomy or implantation of ventilation tubes) by Bretlau et al. and Thomsen et al. as no differences between the groups were noted. Multiple histological studies including a study by Chung et al. refute the procedure of ESD stating that instead of reducing hydrops, post-ESD diffuse hydrops was noted in the cochlea, the saccule, the utricle, and the ampulla [13].

The failure of all these procedures may be due to the inadequate understanding of the pathophysiology of hydrops. A new theory postulated by Saliba et al. states that the organic substrate of the disease surplus of endolymph causing the hydrops also originates in the ES, thus a new technique for Saliba et al. treatment was postulated which is EDB. This technique involves placing a titanium clip over the ED opening thus separating ES from the rest of the inner ear. Studies by Saliba et al. have shown that patients who underwent EDB had a complete recovery from vertigo and a good quality of life [14]. Thus, it confers EDB as the most effective surgical management of MD till now.

Even though EDB is considered the surgery of choice, the identification of the ED opening is sometimes found to be tedious and difficult, especially for beginners. No new technique apart from the usage of the Donaldson line to identify the ES and then the dissection of bone over the vestibular operculum to identify the ED is currently in place. Shea et al. state that the Donaldson line failed to identify the ES in many instances, and he used the otic capsule of the posterior semicircular canal and the tip of the short process of incus to identify the ES [15]. In our study, we are discussing the novel technique for the identification of ED using the MCF dural plate as a reference point and then the angle made by the MCF dural plate and ED in the cadaveric dissection of 20 wet temporal bones. In order to identify the duct in its superior and inferior half in continuity from the ES, the ED is typically found by first identifying the ES using the Donaldson line and then dissecting the bone of the VA operculum and the posterior fossa dura from the retrolabyrinthine bone. To clip the duct, a space is made for the instrument tips to be inserted [8]. In our study, we kept the MCF dural plate as a reference point in all temporal bones, and identification of ED was made feasible by drawing the “GauthamaPrasads” line. Angles made by the “GauthamaPrasads” line (MCF dural plate to ED) were calculated in all the 20 wet temporal bones, which range between 36.00° and 45.99° and were documented (Table 1). The most common angles were 36.00º and 42.99º. We also observed that the GP line is more inferiomedially located compared to that of the Donaldson line, which further states that the endolymphatic opening is more inferiomedially located compared to that of the Donaldson line. Thus, in the future along with the Donaldson line, the otic capsule of the posterior semicircular canal, and the tip of the short process of incus used for identification of ED, the MCF dural plate will also be considered for accurate identification of ED opening, thus endless blind drilling to find the ED opening can be avoided and the time taken for the surgery will be greatly reduced. MCF dural plate can be considered a reliable and constant landmark for identification of ED opening.

Even though as a pilot study still we could find a constant angulation and a reference range for the identification of ED in our dissected bones, anatomical variations can occur, thus research with a larger sample and application of this landmark in real patients undergoing ESB is required in the future for standardizing the procedure.

## Conclusions

Surgical identification of the ED using MCF as a reference point is feasible with a GP line which makes an angulation of 36.00°-45.99° with an MCF dural plate. Accurate identification of the ED was feasible using this new anatomical landmark. These findings provide valuable anatomical insights that can aid in more efficient and accurate surgical interventions involving the ED.
